# Signal propagation in small networks of Hodgkin-Huxley neurons

**DOI:** 10.3389/fnetp.2025.1729999

**Published:** 2025-12-02

**Authors:** Tatiana R. Bogatenko, Konstantin S. Sergeev, Galina I. Strelkova

**Affiliations:** Department of Radiophysics and Nonlinear Dynamics, Institute of Physics, Saratov State University, Saratov, Russia

**Keywords:** Hodgkin-Huxley neuron, synchronization, neural oscillations, neural networks, network physiology

## Abstract

The study of neuron models and their networks is a riveting topic for many researchers worldwide because it allows to glimpse the fundamental processes using accessible methodology. The paper considers dynamics of small networks of Hodkin-Huxley neurons, namely a chain of three neurons and a small-world-like network of seven neurons. The ensembles of neurons are represented by systems of ordinary differential equations, so the research has been conducted numerically. It has been found that complex quasi-periodic and chaotic regimes may arise in the systems, and the existense of such regimes is caused by the inner parameters of the systems, such as individual currents of the neurons and the coupling between them. This research contributes to the fundamental understanding of signal propagation in networks of neuron models and may provide insight into the physiology of real neuronal systems.

## 1 Introduction

Specialists from a wide range of scientific fields are interested in the processes occurring in the brain. Attempts to understand the human brain’s functioning increasingly incorporate concepts from physics, mathematics, computer science, mathematical biology, and related disciplines. So, the development of synergetic approaches (Haken, 1977) has provided a new perspective which can advance the investigation of such processes.

A significant number of fundamental interdisciplinary studies have been conducted by *Hermann Haken*, one of the founders of synergetics. A general description of the brain’s operational principles from a synergetics standpoint is detailed in (Haken, 2013) and the works cited therein, while there is a number of works that inquire about specific phenomena. For instance, some works cover the issue of spontaneous synchronization of neuronal spiking (Haken, 1977; Osipov et al., 2007), and the work (Haken, 1983) is devoted to the competition between oscillatory modes in neural networks and the emergence of a dominant mode.

*Synergetics*’ methodology and approaches have evolved significantly over the past decades, and modern science has come closer to understanding the functioning principles of the brain. However, the brain is a very complex object, and often we can only model a small part of it or are forced to be restricted with general small-world models (Watts and Strogatz, 1998).

Many research groups suggest phenomenological brain models of different complexity in order to understand its general behavior (Dimulescu et al., 2025; Cakan and Obermayer, 2020; Rosenblum, 2024). However, because these models are not directly grounded in the macroscopic characteristics of real neurons, key dynamical differences may exist between the numerical models and actual biological neurons.

Many studies exploring the dynamics of complex brain networks tend to utilize abstract neuron models, for example FitzHugh–Nagumo or integrate-and-fire models, as their partial elements (Rybalova et al., 2023; Wang et al., 2021; Anesiadis and Provata, 2022; Tyloo, 2024; Augustin and Obermayer, 2017). This preference is generally motivated by the relative simplicity of such systems: they often incorporate no more than three dynamic variables, are dimensionless, and are therefore easier to solve numerically.

In contrast, the Hodgkin–Huxley neuron (Hodgkin and Huxley, 1952) is a physiologically plausible model of spike generation, as it is founded on a macroscopic description of the neuronal membrane’s dynamics, which allows the researcher to draw parallels with parameters from real systems. This leads us to believe that the dynamics of networks constructed with Hodgkin–Huxley neurons are worthy of their own study.

Many works about the Hodgkin–Huxley networks consider random coupling structures (Majhi et al., 2025), which clearly have a high degree of similarity to the structures in a real brain. However, studying such structures poses a number of challenges, both technical and interpretative: such topology requires a large number of calculations and it is unclear which characteristics of the resulting system are suitable for drawing parallels with the real brain. In the presented paper we consider small, elementary structures consisting of only a few Hodgkin-Huxley neuron models with diffusive coupling (Sun et al., 2008). This topology is similar to the so-called ”small world network” concept with short distanse between the nodes, high clustering coefficient and connections through hub.

According to the principles of synergetics, the foundation of self-organization is the emergence of a new order and the increase in complexity of systems through random deviations in the states of their elements and subsystems. Such fluctuations are usually neutralized through negative feedback loops, which ensure the preservation of the structure and the system’s near-equilibrium state. However, in more complex open systems, due to the influx of energy from outside, deviations increase over time causing the effect of collective behavior of elements and subsystems. Ultimately, this process leads either to the destruction of the previous structure or to the emergence of a new order. In this work, we set the goal of understanding how simple neuron-like structures of Hodgkin-Huxley models behave, in order to further generalize the acquired knowledge to more complex nonequilibrium systems and move on to nonequilibrium ensembles composed of such elementary subnetworks. So, we dedicate this article to the memory of *Hermann Haken*, the founder of the *Synergetics*, who was actively involved in the study of the most complex processes occurring in the brain.

The paper is organised as follows. Section 2 introduces the model under consideration, explains its characteristics and describes the design of the numerical experiments. Section 3 presents the results of the experiments in full detail. Its Subsection 3.1 describes the results of the experiments for a chain of three coupled Hodgkin-Huxley neurons, while the Subsection 3.2 provides insight into the dynamics of a small network of seven coupled Hodgkin-Huxley neurons. Section 4 discusses the weaknesses and the prospects of the research, and Section 5 summarizes the findings.

## 2 Model and methods

This paper focuses on the regime formation and signal propagation in small ensembles of Hodgkin-Huxley neurons (Hodgkin and Huxley, 1952). The ensembles are defined by the following set of equations:

$$\begin{array}{cc} \frac{dx_{i}}{dt} & =\frac{1}{C_{m}}\left({\bar{g}}_{K}n^{4}\left(x_{i}-x_{K}\right)+{\bar{g}}_{Na}m^{3}h\left(x_{i}{-}_{Na}\right)+\right.\\ & \left.+{\bar{g}}_{l}\left(x_{i}-x_{l}\right)+I_{ext_{i}}\right)+\overset{N}{\underset{j=0,j\neq i}{\sum }}w_{ij}\left(x_{j}-x_{i}\right),\\ \frac{dn_{i}}{dt} & =\alpha_{n_{i}}\left(x_{i}\right)\left(1-n_{i}\right)-\beta_{n_{i}}\left(x_{i}\right)n_{i}.\\ \frac{dm_{i}}{dt} & =\alpha_{m_{i}}\left(x_{i}\right)\left(1-m_{i}\right)-\beta_{m_{i}}\left(x_{i}\right)m_{i},\\ \frac{dh_{i}}{dt} & =\alpha_{h_{i}}\left(x_{i}\right)\left(1-h_{i}\right)-\beta_{h_{i}}\left(x_{i}\right)h_{i}. \end{array}$$ (1)

Here, the first equation describes the dynamics of the neuron membrane potential x , dependent on the ion current flowing through ion channels in the membrane. The next three equations are responsible for the amount of open ion channels, regulating the ion currents. The α(x) and β(x) functions along with all the other parameters ( $C_{m}=1\;\mu$ F/ ${cm}^{2}$ , $x_{K}=+12$ mV, $x_{Na}=-115$ mV, $x_{l}=-10.63$ mV, ${\bar{g}}_{K}=36$ mmho/ ${cm}^{2}$ , ${\bar{g}}_{Na}=120$ mmho/ ${cm}^{2}$ and ${\bar{g}}_{l}=0.3$ mmho/ ${cm}^{2}$ ) are taken directly from the original paper (Hodgkin and Huxley, 1952).

The sum $\sum_{j\neq i}^{N}w_{ij}\left(x_{j}-x_{i}\right)$ in (Equation 1) introduces linear electric coupling with $w_{ij}$ determining coupling strength. The terms $w_{ij}$ are portrayed as elements of coupling matrix W , which allows one to determine any kind of network topology with ease.

**FIGURE 1:**
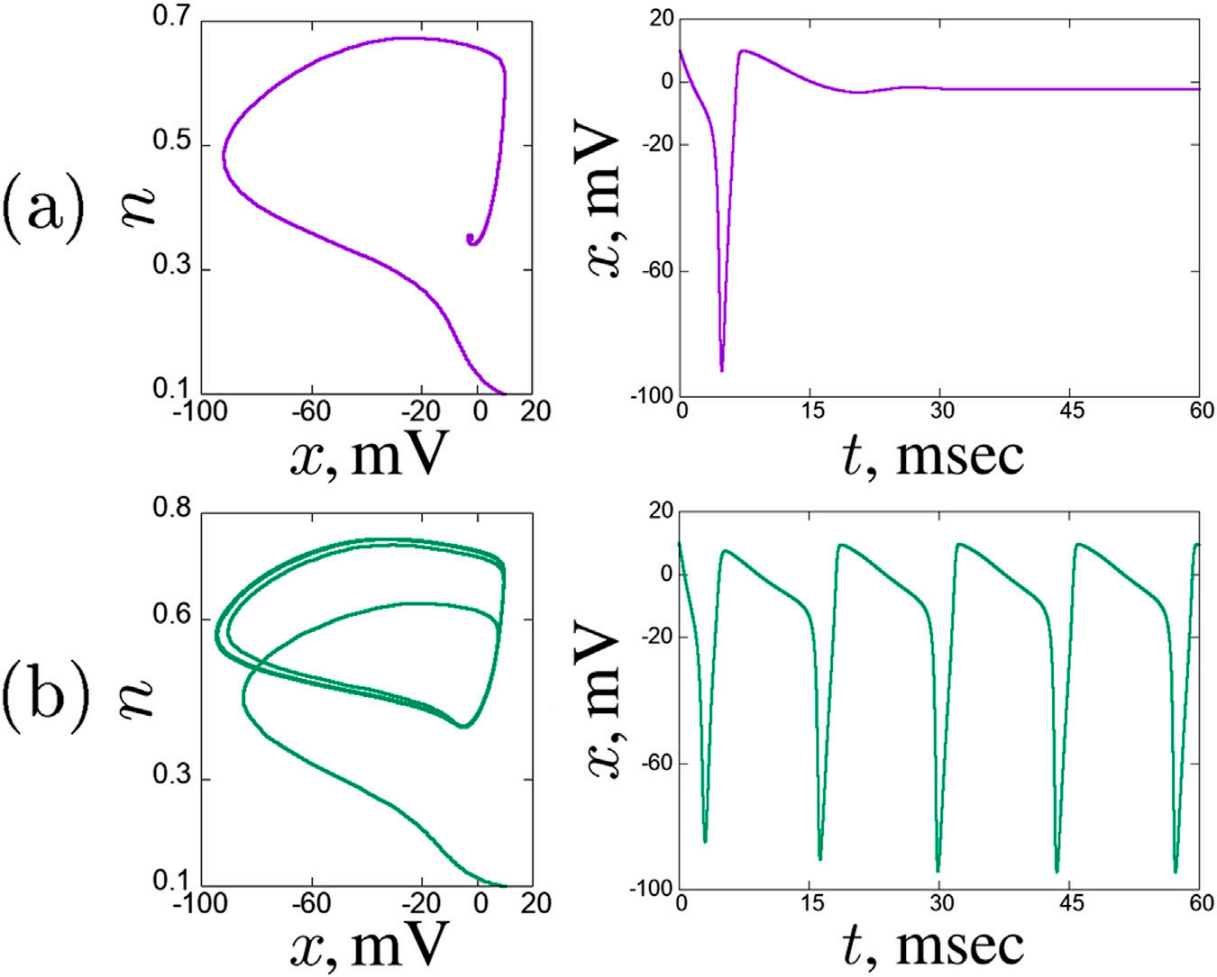
Dynamical regimes in a solitary Hodgkin-Huxley neuron, projections of the phase space on the (x,n) plane (left column) and x(t) time realisations (right column). Excitatory regime for $I_{\mathrm{ext3}}$ = 3 μ A/ ${cm}^{2}$ **(a)** and self-oscillating regime for $I_{\mathrm{ext3}}$ = 12 μ A/ ${cm}^{2}$ **(b)**.

It is well-known that the value of the external current density $I_{\mathrm{ext}}$ affects the change of the regime in a solitary Hodgkin-Huxley system (Hodgkin and Huxley, 1952; Rinzel and Miller, 1980), and the neuron can switch to one of three regimes. In the original Hodgkin-Huxley system, at values of $I_{\mathrm{ext}}\leq 0\mu$ A/ ${cm}^{2}$ oscillations are hindered and the neuron is silent; at $0<I_{\mathrm{ext}}<8\mu$ A/ ${cm}^{2}$ the system shows the excitatory regime and there is a stable focus on the projection of the phase portrait in the x(n) plane (Figure 1a). But at $I_{\mathrm{ext}}=8\mu$ A/ ${cm}^{2}$ , a supercritical Andronov-Hopf bifurcation occurs and the neuron demonstrates self-oscillations, so a stable limit cycle arises on the projection of the phase space in the x(n) plane (Figure 1b).

In this paper we aim to establish the influence of external current densities $I_{\mathrm{ext}}$ and values of coupling strength w on the formation of various dynamical regimes and their propagation within a chain of three and a small ensemble of seven Hodgkin-Huxley neurons. Thereby, the initial conditions had to be fixed at certain values described in the following sections in order to exclude their influence on the dynamics of the considered ensembles. For an ensemble of two Hodgkin-Huxley neurons it has been shown that the regime can depend on the $x_{0}$ initial conditions, while the other initial conditions $n_{0}$ , $m_{0}$ and $h_{0}$ have no influence on the regime formation in the system (Bogatenko et al., 2025).

In order to estimate the synchrony in a chain of three elements, Pearson correlation coefficient (Equation 2) (Pearson, 1896; Dunn and Clark, 1986; Rodgers and Nicewander, 1988) has been used:

$$\rho =\frac{\sum \left(x_{1i}-\bar{x_{1}}\right)\left(x_{2i}-\bar{x_{2}}\right)}{\sqrt{\sum {\left(x_{1i}-\bar{x_{1}}\right)}^{2}\sum {\left(x_{2i}-\bar{x_{2}}\right)}^{2}}}$$ (2)

where $x_{1i}$ and $x_{2i}$ are the elements of the compared series $x_{1}$ and $x_{2}$ , and $\bar{x_{1}}$ and $\bar{x_{2}}$ are the mean values of the series $x_{1}$ and $x_{2}$ . The Pearson correlation coefficient takes values in the range [−1;1] , where 1 means perfect positive or in-phase correlation, −1 means perfect out-of-phase correlation, and 0 means no correlation between the variables. The computation of the correlation coefficient was carried out for complete time realizations. Transient time periods were included in the calculations because they may contain critical information about the regime, for instance, single spikes in excitatory regime.

The research is conducted numerically utilising Runge-Kutta 4th order method (Runge, 1895; Kutta, 1901) over a time interval T=40000 with a step of 0.01 for each time realisation. A set of programs in C was used to carry out numerical integration, and the graphs were plotted with Gnuplot.

## 3 Results

### 3.1 Dynamical effects in a chain of three coupled Hodgkin-Huxley neurons

**FIGURE 2:**
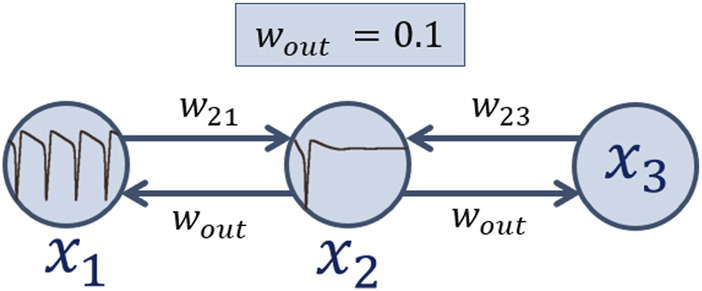
Schematic image of an ensemble of three Hodgkin-Huxley neurons under consideration.

In this section a chain of three coupled Hodgkin-Huxley neurons of the topology shown in Figure 2 is under consideration. Here, the first neuron $x_{1}$ receives $I_{\mathrm{ext1}}=12\mu$ A/ ${cm}^{2}$ and self-oscillates, the second one $x_{2}$ shows excitatory mode for $I_{\mathrm{ext2}}=3\mu$ A/ ${cm}^{2}$ , and $I_{\mathrm{ext3}}$ for the third neuron $x_{3}$ is varied. Also, the second neuron $x_{2}$ always influences the neurons $x_{1}$ and $x_{3}$ with a deliberately low value of coupling strength $w_{\mathrm{out}}=0.1$ , while $w_{21}=w_{23}=w$ are varied. Thus, we analyze the influence of external current density $I_{ext_{3}}$ and coupling strength w on the dynamical regimes and their synchrony in this chain. $I_{ext_{3}}$ were changed in the interval [-15; 15] μ A/ ${cm}^{2}$ , and w took values in [0; 4]. Initial conditions are the same for all the neurons: $x_{01}=x_{02}=x_{03}=10$ , $n_{01}=n_{02}=n_{03}=0.1$ , $m_{01}=m_{02}=m_{03}=0.01$ , $h_{01}=h_{02}=h_{03}=0.01$ , which allows us to exclude their influence on the system’s dynamics.

**FIGURE 3:**
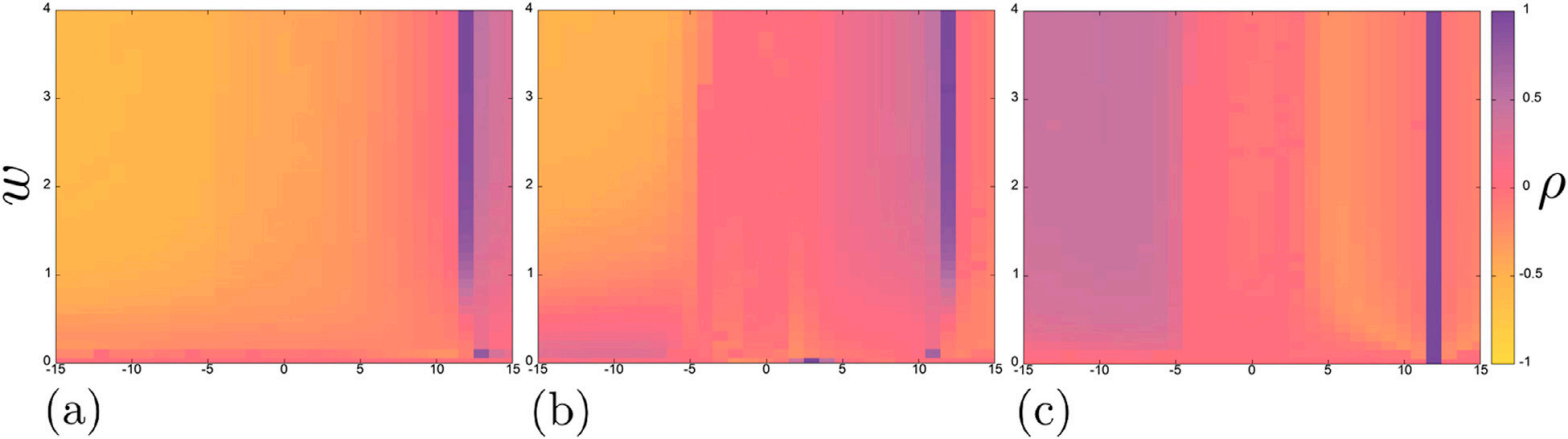
Pearson correlation coefficient maps on the plane $\left(I_{ext_{3}},w\right)$ of the three pairs of neurons in a chain: $x_{1}$ and $x_{2}$ **(a)**, $x_{2}$ and $x_{3}$ **(b)**, $x_{1}$ and $x_{3}$ **(c)**. Other parameters and initial conditions: $I_{ext_{1}}=12\mu$ A/ ${cm}^{2}$ , $I_{ext_{2}}=3\mu$ A/ ${cm}^{2}$ , $x_{01}=x_{02}=x_{03}=10$ , $n_{01}=n_{02}=n_{03}=0.1$ , $m_{01}=m_{02}=m_{03}=0.01$ , $h_{01}=h_{02}=h_{03}=0.01$ .

**FIGURE 4:**
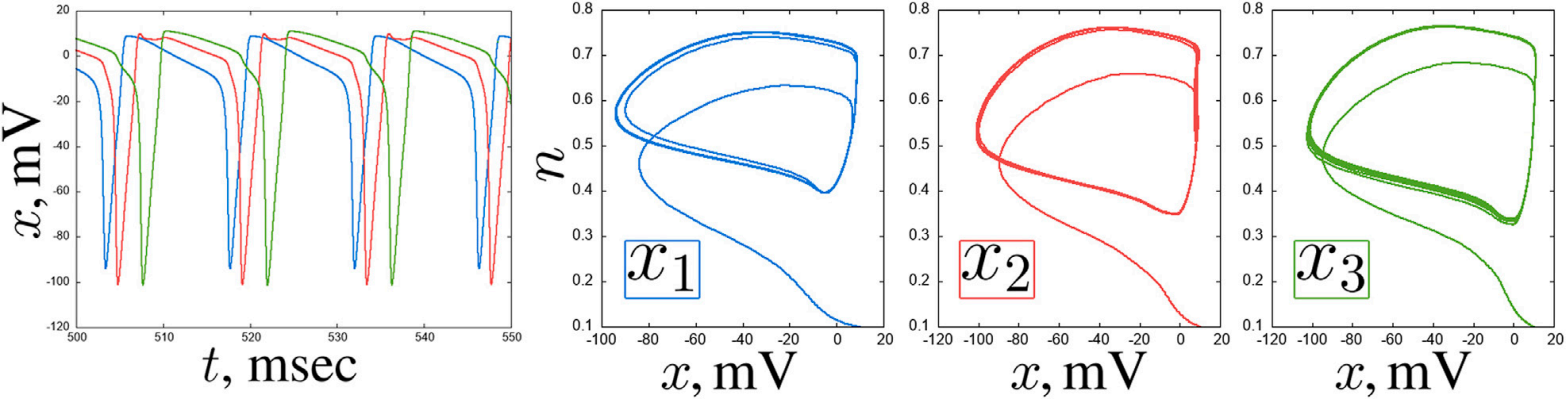
An example of delay in a chain of three coupled Hodgkin-Huxley neurons for $I_{ext_{3}}=1.0\mu$ A/ ${cm}^{2}$ and w=0.2 : x(t) time realisations and projections of the phase space on the (x,n) plane of the neurons $x_{1}$ (blue), $x_{2}$ (red) and $x_{3}$ (green). Other parameters and initial conditions: $I_{ext_{1}}=12\mu$ A/ ${cm}^{2}$ , $I_{ext_{2}}=3\mu$ A/ ${cm}^{2}$ , $x_{01}=x_{02}=x_{03}=10$ , $n_{01}=n_{02}=n_{03}=0.1$ , $m_{01}=m_{02}=m_{03}=0.01$ , $h_{01}=h_{02}=h_{03}=0.01$ .

**FIGURE 5:**
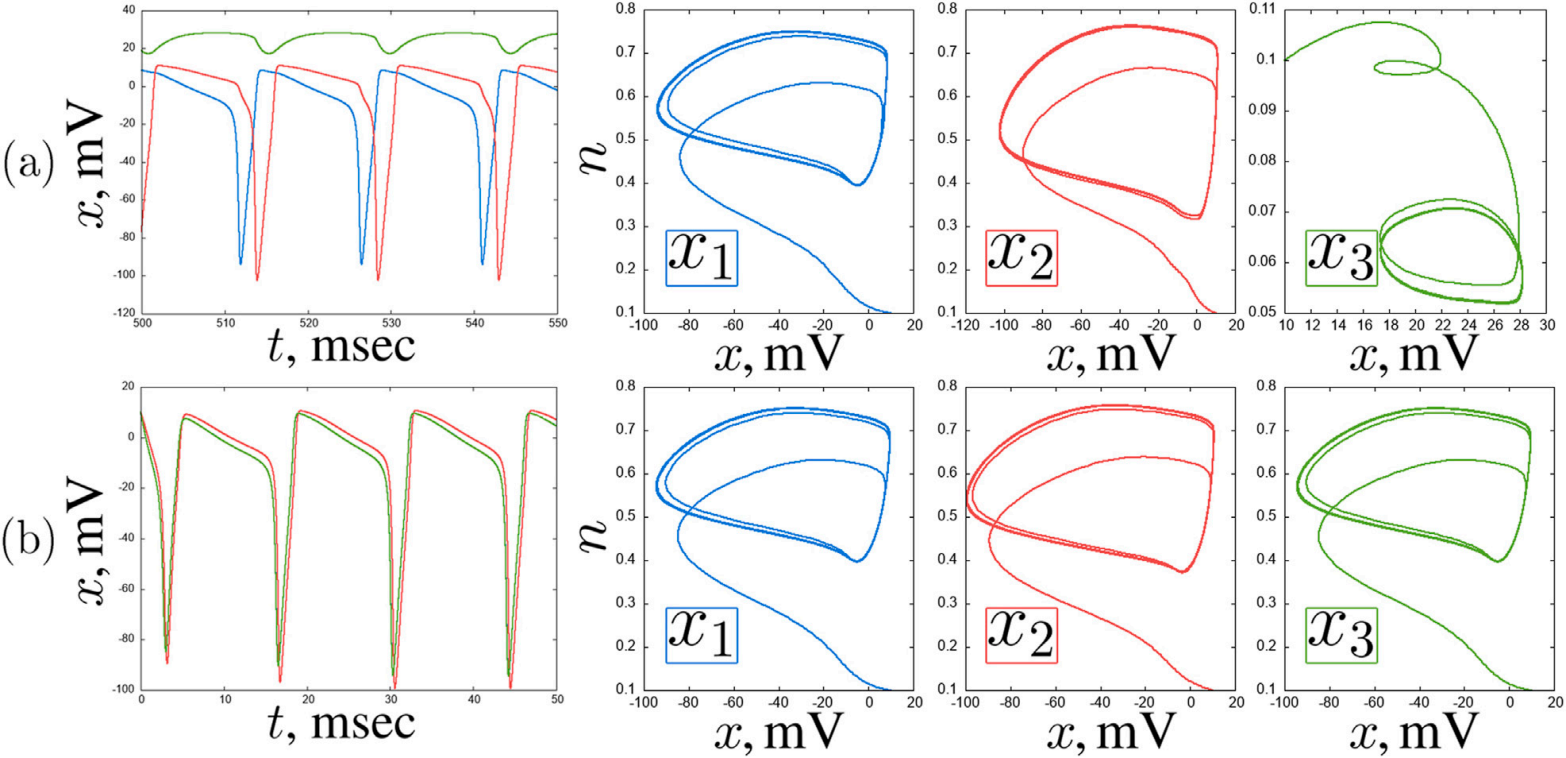
An example of self-oscillating regimes in a chain of three coupled Hodgkin-Huxley neurons for $I_{ext_{3}}=-14.0\mu$ A/ ${cm}^{2}$ , w=0.2 **(a)** and $I_{ext_{3}}=12.0\mu$ A/ ${cm}^{2}$ , w=0.7 **(b)**. x(t) time realisations and projections of the phase space on the (x,n) plane of the neurons $x_{1}$ (blue), $x_{2}$ (red) and $x_{3}$ (green). Other parameters and initial conditions: $I_{ext_{1}}=12\mu$ A/ ${cm}^{2}$ , $I_{ext_{2}}=3\mu$ A/ ${cm}^{2}$ , $x_{01}=x_{02}=x_{03}=10$ , $n_{01}=n_{02}=n_{03}=0.1$ , $m_{01}=m_{02}=m_{03}=0.01$ , $h_{01}=h_{02}=h_{03}=0.01$ .

**FIGURE 6:**
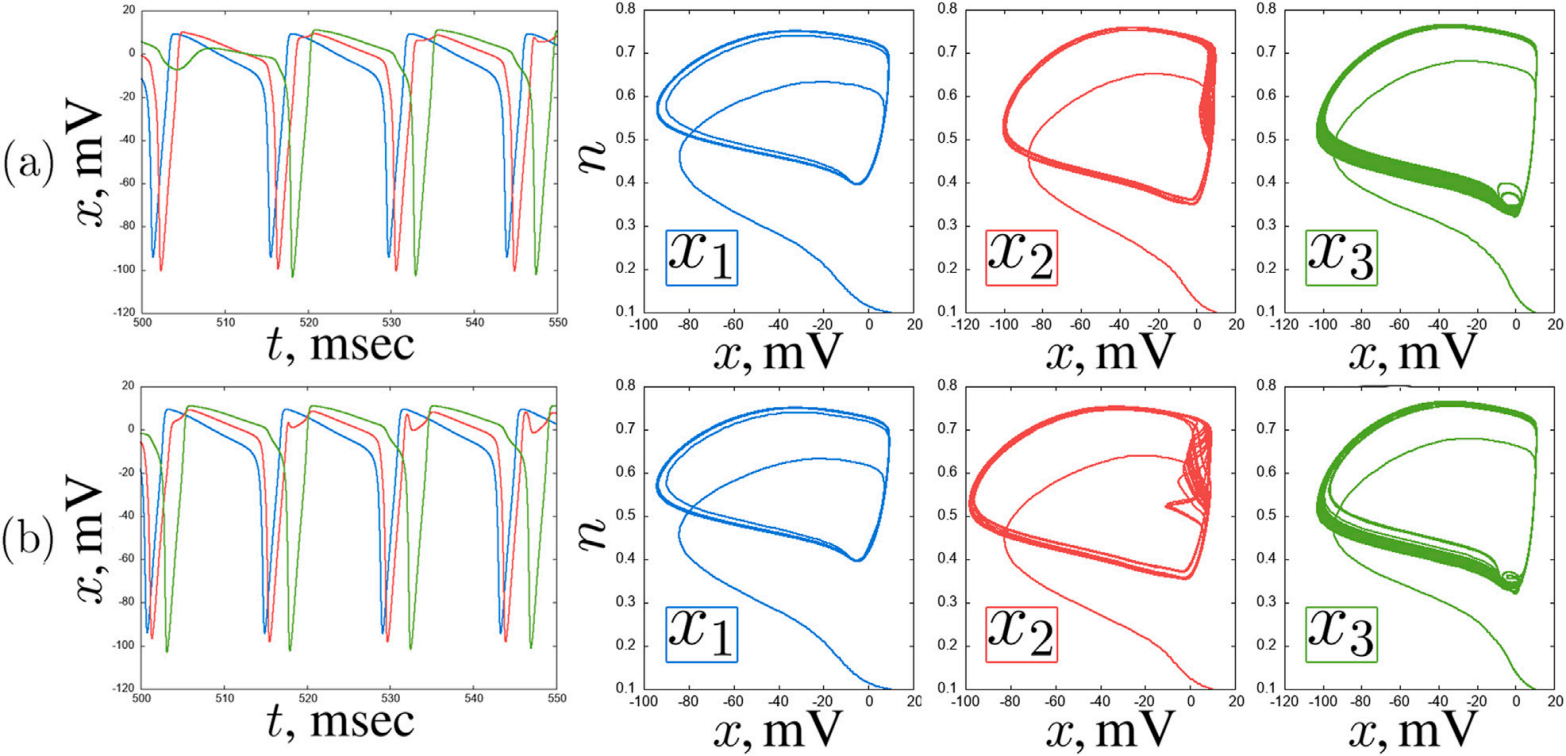
An example of quasiperiodic regimes in a chain of three coupled Hodgkin-Huxley neurons for $I_{ext_{3}}=1.0\mu$ A/ ${cm}^{2}$ , w=0.4 **(a)** and $I_{ext_{3}}=1.0\mu$ A/ ${cm}^{2}$ , w=0.9 **(b)**. x(t) time realisations and projections of the phase space on the (x,n) plane of the neurons $x_{1}$ (blue), $x_{2}$ (red) and $x_{3}$ (green). Other parameters and initial conditions: $I_{ext_{1}}=12\mu$ A/ ${cm}^{2}$ , $I_{ext_{2}}=3\mu$ A/ ${cm}^{2}$ , $x_{01}=x_{02}=x_{03}=10$ , $n_{01}=n_{02}=n_{03}=0.1$ , $m_{01}=m_{02}=m_{03}=0.01$ , $h_{01}=h_{02}=h_{03}=0.01$ .

Figure 3 shows the Pearson correlation coefficient maps for each of the three pairs of neurons in the chain, while Figures 4–6 show some typical time realizations of x(t) and projections of the phase portraits onto the (x,n) plane. In general, one can note that the absolute value of correlation coefficient ρ is equal or close to one for $x_{1}$ and $x_{2}$ on the most of the considered parameter plane, while it is generally closer to 0 for the other pairs. It means that the neurons $x_{1}$ and $x_{2}$ often show correlated behaviour, however, the negative value of the coefficient indicates antiphase oscillations. In all, the presence of areas with negative correlation coefficient values on the three maps is caused by a delay occurring in the neurons (Figure 4).

Besides, on each of the maps in Figure 3a vertical region of complete positive correlation (ρ=1) is noticeable for $I_{\mathrm{ext3}}$ = 12 μ A/ ${cm}^{2}$ , in which the neurons are completely synchronous. The presence of this region precisely at the value $I_{\mathrm{ext3}}$ = 12 μ A/ ${cm}^{2}$ is due to the fact that the first neuron receives an external current of exactly this magnitude: $I_{\mathrm{ext1}}$ = 12 μ A/ ${cm}^{2}$ , and the self-oscillations arising in it suppress the excitable mode of the second neuron ( $I_{\mathrm{ext2}}$ = 3 μ A/ ${cm}^{2}$ ). Note that the suppression of the excitable mode occurs smoothly in terms of the coupling strength w : in the absence of coupling, as well as at small coupling strength values w≪1 , the correlation coefficient is close to zero and the neurons are not synchronous. Then, with an increase in the coupling strength, the correlation coefficient begins to increase and at w≈1.5 it becomes equal to 1 (Figure 3). This effect has been shown earlier for two coupled Hodgkin-Huxley neurons (Bogatenko et al., 2025). On the correlation map of neurons $x_{1}$ and $x_{3}$ (Figure 3c), the coefficient ρ equals to 1 for all the values of coupling strength in the considered range at $I_{\mathrm{ext3}}$ = 12 μ A/ ${cm}^{2}$ , since in this case these two neurons show the same regime throughout the experiment.

What the correlation maps in Figure 3 do not show is boundaries between oscillation modes. However, as shown in some representative examples, the neurons tend to exhibit oscillations of varying complexity throughout the entire parameter plane under consideration. Self-oscillations of classical spikes can be synchronized with high accuracy (ρ=1) (Figure 5b), but there also are oscillations that resemble spikes in shape but have a much smaller amplitude of up to 5 mV (Figure 5a). Also, quasi-periodic oscillation regimes of varying degrees of complexity can be realized in the system (Figure 6).

### 3.2 Signal propagation in a network of seven coupled Hodgkin-Huxley neurons

**FIGURE 7:**
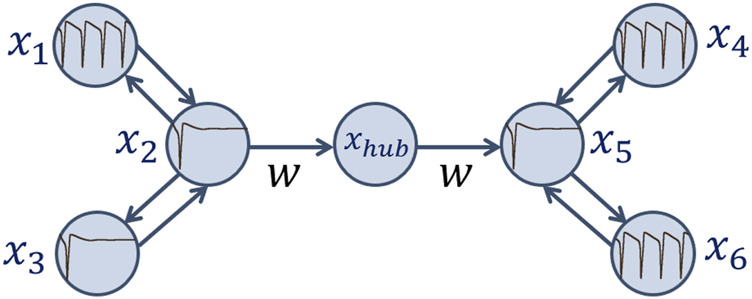
Schematic image of an ensemble of seven Hodgkin-Huxley neurons under consideration.

Now let us construct a larger network of Hodgkin-Huxley neurons, consisting of two smaller chains connected unidirectionally via a hub (Figure 7). Within the chains, the neurons are connected bidirectionally, and the connection values between them remain fixed in all numerical experiments.

**FIGURE 8:**
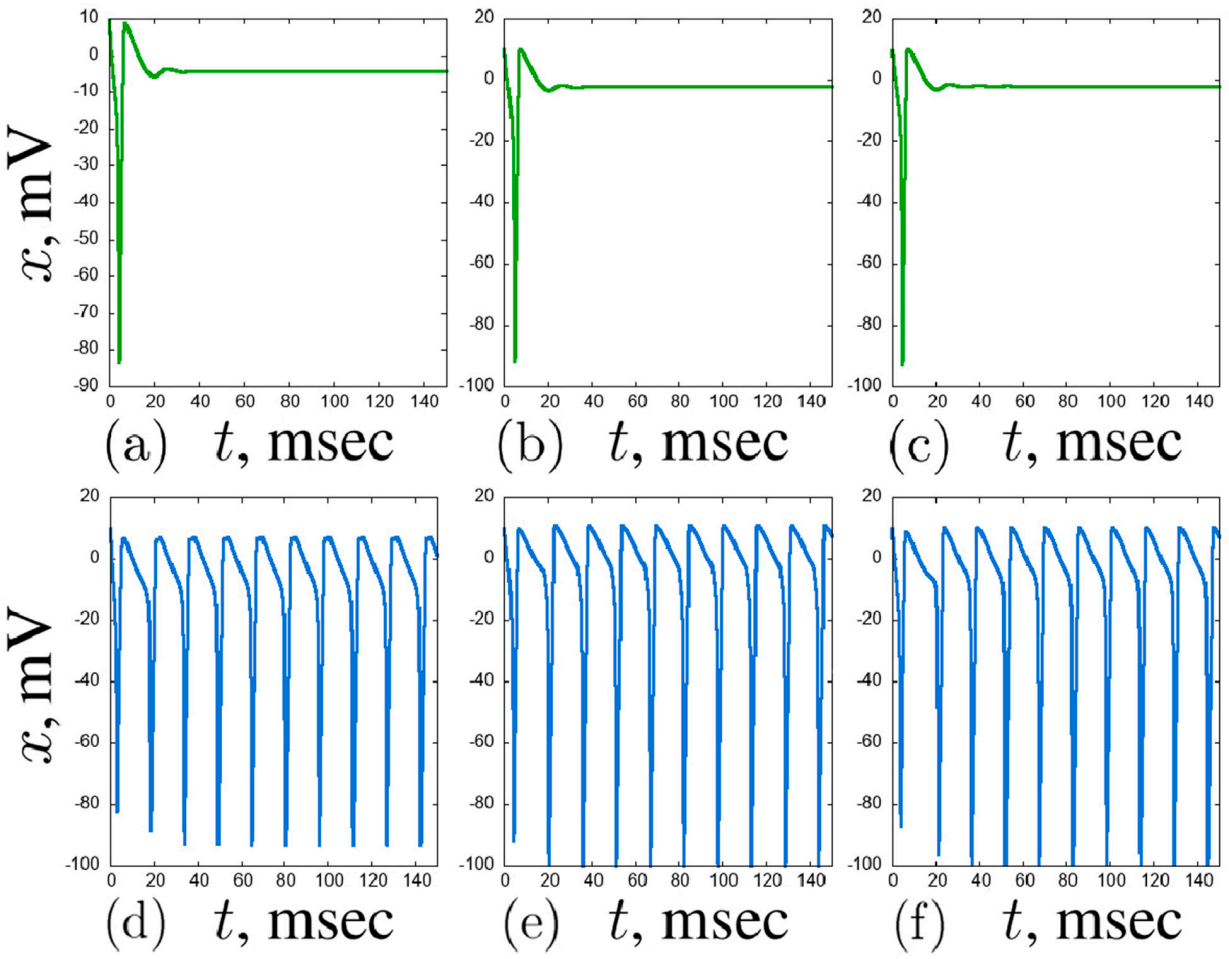
Structures in the first (green) and the second (blue) layer of the considered network of seven Hodgkin-Huxley neurons in each experiment: x(t) time realisations of $x_{1}$ **(a)**, $x_{2}$ **(b)**, $x_{3}$ **(c)**, $x_{4}$ **(d)**, $x_{5}$ **(e)**, and $x_{6}$ **(f)**. Parameters: $I_{\mathrm{ext1}}$ = $I_{\mathrm{ext4}}$ = 12 μ A/ ${cm}^{2}$ , $I_{\mathrm{ext2}}$ = $I_{\mathrm{ext5}}$ = 3 μ A/ ${cm}^{2}$ , $I_{\mathrm{ext3}}$ = 2 μ A/ ${cm}^{2}$ , $I_{\mathrm{ext6}}$ = 7 μ A/ ${cm}^{2}$ ; $w_{21}=w_{23}=0.1$ , $w_{12}=w_{32}=2.5$ ; $w_{54}=w_{56}=0.1$ , $w_{45}=w_{65}=0.3$ .

At the beginning of each experiment, in the first chain one of the outer neurons is in the self-oscillation mode ( $I_{\mathrm{ext1}}$ = 12 μ A/ ${cm}^{2}$ ), and the other two show the excitatory mode, but with a small detuning in the value of the external current density: $I_{\mathrm{ext2}}$ = 3 μ A/ ${cm}^{2}$ , $I_{\mathrm{ext3}}$ = 2 μ A/ ${cm}^{2}$ . Also, the outer neurons intentionally exert a weak influence on the central neuron $\left(w_{21}=w_{23}=0.1\right)$ , while in the opposite direction, the coupling strength is deliberately set high – $w_{12}=w_{32}=2.5$ . Such a combination of external currents and coupling strength values forces a certain regime to establish in this layer: all the three neurons are nearly synchronous in the excitatory mode (Figures 8a–c).

In the second chain, there is a similar balance of connection strengths as in the first one, but here the central neuron acts on the outer neurons with a weaker coupling strength compared with the first layer: $w_{54}=w_{56}=0.1$ , $w_{45}=w_{65}=0.3$ . Here, similar regimes are realized in the neurons: $I_{\mathrm{ext4}}$ = 12 μ A/ ${cm}^{2}$ , $I_{\mathrm{ext5}}$ = 3 μ A/ ${cm}^{2}$ , $I_{\mathrm{ext6}}$ = 7 μ A/ ${cm}^{2}$ , however, we note that the current density $I_{\mathrm{ext6}}$ received by neuron $x_{6}$ is of a prethreshold value for the Andronov-Hopf bifurcation. Now, self-oscillating regime establishes in this chain at the beginning of every experiment (Figures 8d–f).

Here we aspire to establish the influence of the coupling strength between the layers through the hub and the hub’s regime on the dynamics that is translated to the second chain. This goal motivates the choice of constant values of the coupling strength and the current density values within the chains. For the initial conditions in the system in all the experiments described in this section, a single set of values with a uniform distribution over the intervals [9.5;10.5] was chosen for the set of seven variables x , [0.095;0.105] for the set of seven variables n , and [0.0095;0.0105] for the sets of variables m and h (seven values each). The initial conditions were chosen in this way in order to introduce a degree of dissimilarity and bring the numerical experiment closer to real systems.

**FIGURE 9:**
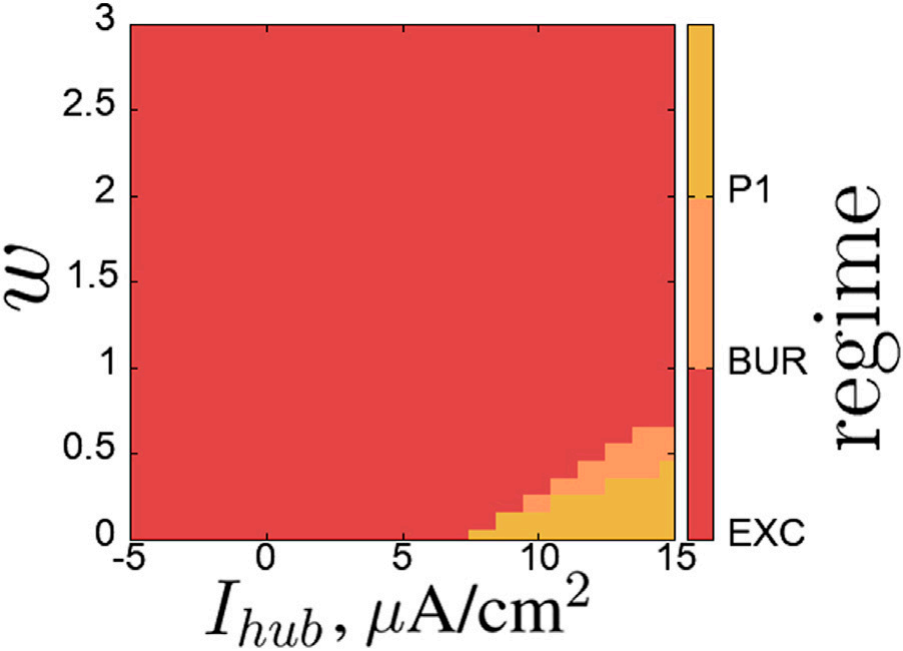
Regime map of the hub neuron $x_{\mathrm{hub}}$ of the considered network of seven Hodgkin-Huxley neurons on the parameter plane $\left(I_{\mathrm{hub}},w\right)$ . Regime scale: EXC–excitatory regime, BUR–single burst with subsequent silence, P1 – periond-1 oscillations. Parameters are stated in Section 3.2.

**FIGURE 10:**
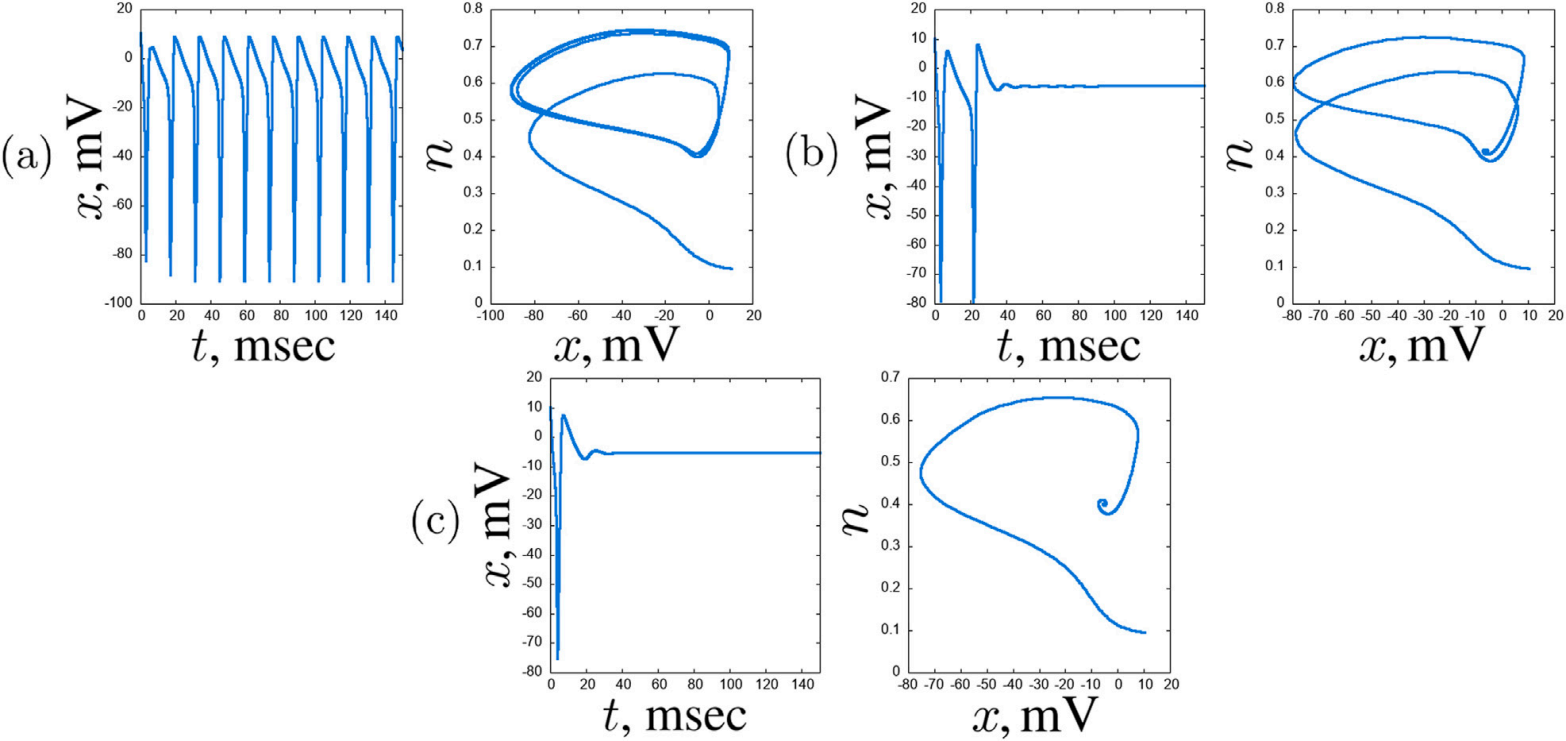
An example of typical regimes in the hub neuron $x_{\mathrm{hub}}$ of the considered network of seven Hodgkin-Huxley neurons: x(t) time realisations and projections of the phase space on the (x,n) plane. Period-1 oscillations **(a)** ( $I_{\mathrm{hub}}$ = 14 μ A/ ${cm}^{2}$ , w=0.2 ), single burst **(b)** ( $I_{\mathrm{hub}}$ = 14 μ A/ ${cm}^{2}$ , w=0.6 ), and excitatory regime **(c)** ( $I_{\mathrm{hub}}$ = 14 μ A/ ${cm}^{2}$ , w=1.4 ) are shown. Other parameters are stated in Section 3.2.

Figure 9 shows the regime map for the hub neuron on the parameter plane ( $I_{\mathrm{hub}}$ , w ). One can see that the hub neuron can show one of three regimes: excitatory, single burst, and period-1 self-oscillations. Self-oscillations are observed in a limited parameter range with low coupling strength w<0.5 and current density values greater than 7 μ A/ ${cm}^{2}$ (Figure 10a). As the coupling strength increases, the region of self-oscillations decreases—here one can see how the regime established in the first chain suppresses the self-oscillation regime in the hub neuron. The excitatory regime predominates over most of the plane under consideration (Figure 10c). The boundary between these regimes is expressed in the regime of single burst generation (Figure 10b).

**FIGURE 11:**
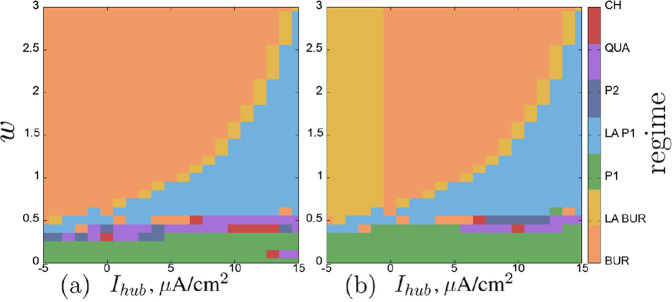
Regime map of the neurons $x_{5}$ **(a)** and $x_{6}$ **(b)** of the second layer of the considered network of seven Hodgkin-Huxley neurons on the parameter plane $\left(I_{\mathrm{hub}},w\right)$ . Regime scale: BUR–single burst with subsequent silence, LA BUR–single burst of low-amplitude oscillations (up to 5 mV), P1 – periond-1 oscillations, LA P1 – low-amplitude self-oscillations (up to 5 mV), P2 – period-2 self-oscillations, QUA–quasi-periodic oscillations, CH–chaotic oscillations. Parameters are stated in Section 3.2.

Regime maps for the neurons in the second layer on the same parameter plane are shown in Figure 11. Within the considered parameter ranges, neurons can exhibit a variety of simpler and more complex regimes: excitatory regime (EXC), single burst with subsequent silence (BUR), single burst of low-amplitude oscillations (up to 5 mV) (LA BUR), period-1 self-oscillations (P1), low-amplitude self-oscillations (up to 5 mV) (LA P1), period-2 self-oscillations (P2), quasi-periodic (QUA), and chaotic (CH) oscillations.

**FIGURE 12:**
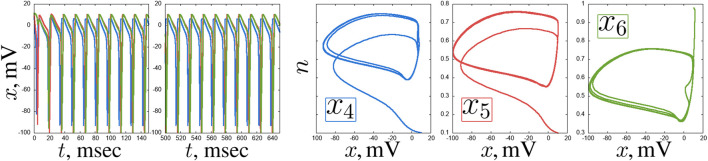
An example of a self-oscillating regime in the second layer of the considered network of seven Hodgkin-Huxley neurons for $I_{\mathrm{hub}}$ = 6 μ A/ ${cm}^{2}$ and w=0.1 : x(t) time realisations and projections of the phase space on the (x,n) plane of the neurons $x_{4}$ (blue), $x_{5}$ (red) and $x_{6}$ (green). Other parameters are stated in Section 3.2.

**FIGURE 13:**
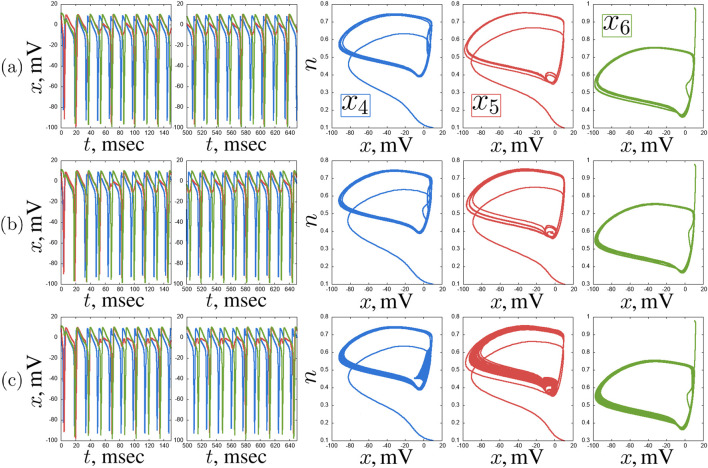
Examples of complex transient regimes in the second layer of the considered network of seven Hodgkin-Huxley neurons: x(t) time realisations and projections of the phase space on the (x,n) plane of the neurons $x_{4}$ (blue), $x_{5}$ (red) and $x_{6}$ (green). Cases for $I_{\mathrm{hub}}$ = 4 μ A/ ${cm}^{2}$ , w=0.4 **(a)**, $I_{\mathrm{hub}}$ = 14 μ A/ ${cm}^{2}$ , w=0.5 **(b)**, and $I_{\mathrm{hub}}$ = 7 μ A/ ${cm}^{2}$ , w=0.5 **(c)** are shown. Other parameters are stated in Section 3.2.

The regime maps for neurons of the second chain $x_{5}$ and $x_{6}$ have a more complex structure than the map for the hub neuron. Here, for small values of the coupling strength with the hub neuron w<0.3 , the self-oscillation mode of period 1 prevails in the system - for these parameter values neuron $x_{4}$ transmits its self-oscillation mode to neurons $x_{5}$ and $x_{6}$ (Figure 12). In the range of values w∈[0.3,0.5] , there is a region of transient modes, when the value of the coupling strength with the hub neuron w becomes greater than the value of the coupling strength within the chain $w_{45}=w_{65}=0.3$ . Here we can observe various complex regimes: period-2 oscillations, quasi-periodic and chaotic oscillations (Figure 13).

**FIGURE 14:**
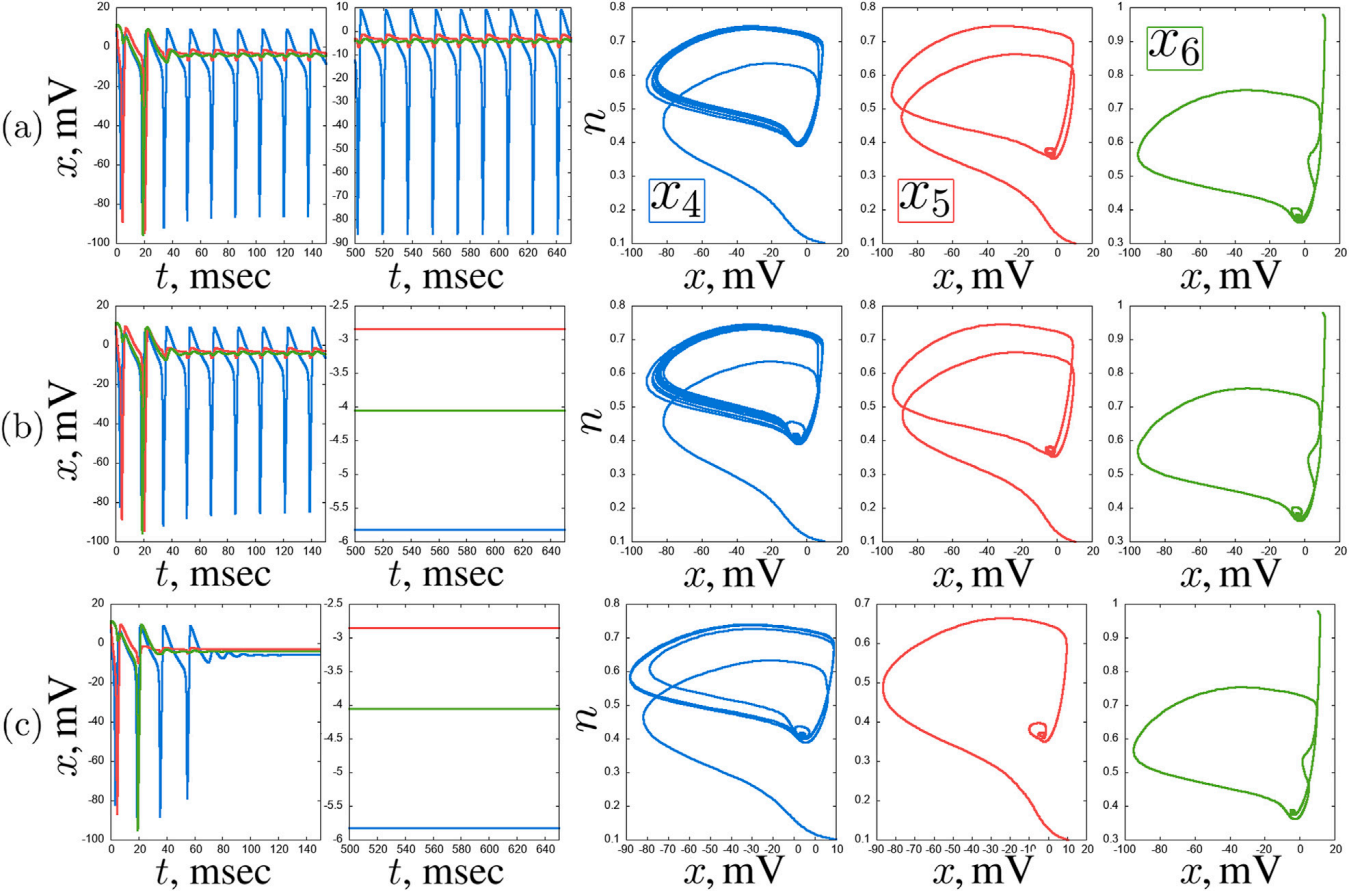
Examples of stable regimes in the second layer of the considered network of seven Hodgkin-Huxley neurons: x(t) time realisations and projections of the phase space on the (x,n) plane of the neurons $x_{4}$ (blue), $x_{5}$ (red) and $x_{6}$ (green). Cases for $I_{\mathrm{hub}}$ = 7 μ A/ ${cm}^{2}$ , w=0.1 **(a)**, $I_{\mathrm{hub}}$ = 7 μ A/ ${cm}^{2}$ , w=1.1 **(b)**, and $I_{\mathrm{hub}}$ = 7 μ A/ ${cm}^{2}$ , w=2.3 **(c)** are shown. Other parameters are stated in Section 3.2.

With further increase in the coupling strength with the hub neuron w , the regime map becomes more regular. Here, one can see the predominance of two modes: period-1 self-oscillations of low amplitude (Figure 14) and single burst with subsequent silence (Figure 14). Low-amplitude period-1 self-oscillations occur with an amplitude smaller than in the classical self-oscillatory mode of the system and are approximately 5 mV. Interestingly, the boundary between these two modes has the form of a hyperbolic or exponential function and is expressed by the mode of generating a low-amplitude burst (Figure 14). It is also interesting to note that for neuron $x_{6}$ , this mode of generating a small-amplitude spike train is not only transient, as for neuron $x_{5}$ , but also a stable mode, which is realized at negative values of the current density $I_{\mathrm{hub}}$ (Figure 9).

## 4 Discussion

The obtained results demonstrate that elementary Hodgkin-Huxley neuronal networks, renowned for their physiological fidelity, are capable of generating a rich repertoire of complex dynamics based solely on their internal structure and connectivity, without the need for complex external driving forces. Our study of two different topologies reveals several fundamental principles that govern the emergence of this complexity.

A key limitation of the current study is its deliberate focus on a set of specific, predetermined network topologies, which, while providing a controlled framework for analysis, may not fully capture the architectural diversity found in biological neural systems. Future research must therefore prioritize scaling these investigations to larger, more complex networks to determine if the observed dynamics–such as the interplay between sodium-channel persistence and delayed-rectifier potassium currents in shaping burst termination–are preserved or fundamentally altered. It will be crucial to examine how these dynamics are influenced by other overarching network characteristics. For instance, introducing a hierarchical organization, where clusters of neurons with specific firing properties are nested within larger functional modules, could reveal novel emergent computational states. Furthermore, the inclusion of adaptive, plastic synapses based on spike-timing-dependent plasticity (STDP) rules would transform the model from a static circuit into a dynamic, learning system. This would allow us to investigate whether the intrinsic neuronal properties governed by the Hodgkin-Huxley formalism interact synergistically or competitively with experience-dependent synaptic changes to guide network development and stability.

The choice of coupling in the model, presented in Equation 1, is also in line with maintaining the scalability of our research to larger and complex networks and is due to two main reasons. Firstly, its mathematical simplicity and well-defined properties provide a clear and interpretable framework for isolating the effects of network topology on the emergent dynamics of intrinsically bursting neurons. This allows us to distinguish effects stemming from the network architecture from those arising from the complexities of synaptic transmission. Secondly, for the purpose of a qualitative analysis of mode generation in elementary neuromorphic structures, diffusion coupling offers a computationally efficient yet biologically grounded foundation. While models incorporating detailed chemical synapses are essential for simulating specific cortical circuits, their complexity would obscure the primary focus of this study: the interplay between topology and intrinsic neuronal dynamics. Certainly, there are numerous ways to introduce coupling between Hodgkin-Huxley neurons [see (Rossoni et al., 2005; Ma and Tang, 2017; Petousakis et al., 2023; Catterall et al., 2012) and many others], including special software (Hines and Carnevale, 1997; Carnevale and Hines, 2006; Bologna et al., 2022; Willms, 2002), all of which are biologically justified to varying degrees. More complex models of coupled Hodgkin-Huxley neurons are typical primarily for modeling specialized cortical regions and parts of the nervous system and, therefore, are beyond the scope of the problem being solved here.

In assessing the biological plausibility of the model, it is important to consider the key output parameters it generates. Characteristic current values range from a few to tens of nanoamperes and fall well within the recognized physiological range for mammalian neurons (Kole and Stuart, 2012; Stuart and Sakmann, 1994). Moreover, the temporal dynamics with spike durations of 1–2 ms accurately reflect those observed in experimental recordings from various cortical and hippocampal cell types (McCormick et al., 1985). The firing rates obtained in our simulations, often in the gamma range (20–80 Hz) under moderate inputs, are possibly related to oscillations associated with cognitive processing (Buzsaki and Draguhn, 2004). Although the model may exhibit higher rates under extreme forcing forces–a known property of the underlying Hodgkin–Huxley formalism, this does not detract from its usefulness for studying the fundamental network dynamics of spikes initiation, propagation, and synchronization, which was the primary goal of this work. Therefore, we are confident that the model provides a physiologically sound platform for investigating the interaction between the intrinsic properties of neurons and network topology.

## 5 Conclusion

In a small chain of coupled Hodgkin-Huxley neurons, we observe that the classical self-oscillatory mode, although dominant, is not exclusive. The emergence of more complex quasi-periodic oscillations and the observed delay in signal propagation along the network underscore a key conclusion: the intrinsic properties of the network itself are the primary source of complexity. Specifically, it is the precise interplay of three factors that regulates these dynamic modes: network topology, neurons’ coupling strength, and the combination of individual neuron current densities. It is important because it suggests that even relatively simple, localized neural circuits harbor a latent capacity to form complex temporal patterns, which may be fundamental to processes such as central pattern generation or sensory processing.

This principle is further reinforced in the small-world ensemble with a hub. Here, the hub neuron acts as a nonlinear processor and integrator, enabling the system to generate not only quasi-periodic, but also chaotic oscillations. Parameter induced transitions between dynamical regimes (silence, periodic, quasi-periodic, and chaotic) have well-defined boundaries in the regime maps for the parameters $I_{\mathrm{hub}}$ and w . But this predictability is non-trivial–it indicates that, despite the potential for chaos, the system’s behavior is determined by an underlying order that can be controlled by specific biological analogs–the excitability of the key neuron and the coupling strength.

More broadly, our results are consistent with the synergetic view of the brain as a self-organizing system. The spontaneous emergence and predictable transitions between complex oscillatory modes, driven by intrinsic network parameters rather than external stimuli, provide a plausible model for the dynamic switching of neural ensembles between functional states. The hub-based model, in particular, offers a mechanistic explanation for how local changes (e.g., neuromodulation affecting $I_{\mathrm{hub}}$ and w ) can lead to global shifts in network dynamics, which may be important for understanding the role of highly connected nodes in neural networks.

## Data Availability

The raw data supporting the conclusions of this article will be made available by the authors, without undue reservation.
